# Non-falciparum malaria in Dakar: a confirmed case of *Plasmodium ovale wallikeri* infection

**DOI:** 10.1186/s12936-016-1485-1

**Published:** 2016-08-24

**Authors:** Mamadou A. Diallo, Aida S. Badiane, Khadim Diongue, Awa Deme, Naomi W. Lucchi, Marie Gaye, Tolla Ndiaye, Mouhamadou Ndiaye, Louise K. Sene, Abdoulaye Diop, Amy Gaye, Yaye D. Ndiaye, Diama Samb, Mamadou S. Yade, Omar Ndir, Venkatachalam Udhayakumar, Daouda Ndiaye

**Affiliations:** 1 Laboratoire de Parasitologie-Mycologie, Université Cheikh Anta Diop de Dakar, Avenue Cheikh Anta Diop, Fann, BP 5005, Dakar, Senegal; 2 Malaria Branch, Division of Parasitic Diseases and Malaria, Center for Global Health, Centers for Disease Control and Prevention, Atlanta, GA USA

**Keywords:** *Plasmodium ovale*, Malaria, Fever, RDT, Microscopy, Diagnostic, Treatment, Primaquine, Dakar

## Abstract

**Background:**

*Plasmodium ovale* is rarely described in Senegal. A case of clinical malaria due to *P. ovale wallikeri* in West Central of Senegal is reported.

**Case:**

A 34-year-old male baker in Dakar, with no significant previous medical history, was admitted to a health clinic with fever and vomiting. Fever had been lasting for 4 days with peaks every 48 h. As monospecific *Plasmodium falciparum* HRP-2 RDT was negative, he was treated with antibiotics. However, owing to persisting symptoms, he was referred to the emergency unit of the Youssou Mbargane Diop Hospital, Dakar, Senegal. Clinical examination found impaired general condition. All other physical examinations were normal. Laboratory tests showed anaemia (haemoglobin 11.4 g/dl), severe thrombocytopaenia (platelets 30 × 10^9^/mm^3^), leukopenia (3650/mm^3^), lymphocytopenia (650/mm^3^). Renal function was normal as indicated by creatininaemia and uraemia (11 mg/l and 0.25 g/l, respectively) and liver enzymes were slightly elevated (aspartate aminotransferase 77 UI/l and alanine aminotransferase 82 UI/l). Blood smear evaluations in Parasitology Laboratory of Aristide Le Dantec Hospital showed malaria parasites of the species *P. ovale* with a 0.08 % parasitaemia. Molecular confirmation was done by real time PCR targeting the 18S rRNA gene. The *P. ovale* infection was further analysed to species level targeting the *potra* gene and was identified as *P. ovale wallikeri*. According to the hospital’s malaria treatment guidelines for severe malaria, treatment consisted of intravenous quinine at hour 0 (start of treatment) and 24 h after initial treatment, followed by artemether–lumefantrine 24 h later. A negative microscopy was noted on day 3 post-treatment and the patient reported no further symptoms.

**Conclusion:**

Malaria due to non-falciparum species is probably underestimated in Senegal. RDTs specific to non-falciparum species and/or pan specific RDTs should be included as tools of diagnosis to fight against malaria in Senegal. In addition, a field-deployable molecular tool such as the loop-mediated isothermal amplification can be considered as an additional useful tool to detect low malaria parasite infections and for speciation. In addition, national malaria control policies should consider other non-falciparum species in treatment guidelines, including the provision of primaquine for the treatment of relapsing parasites.

## Background

Despite the fact that *Plasmodium ovale* is endemic in tropical Africa, malaria due to *P. ovale* is rarely described in Senegal [1]. The most common pathogen of malaria in Africa is *Plasmodium falciparum* [2]. In the 1990s, *P. ovale* infections were described in the south of Senegal in the context of the longitudinal study at Dielmo village located 280 km South East of Dakar [1]. During the first year of the project in 1990, the prevalence of *P. ovale* was determined, among symptomatic and asymptomatic villagers of all groups age, to be 5.5 % either as a mono-infection or as mixed infection with *P. falciparum* [3]. Concerted efforts to control malaria in Senegal contributed to decrease in the overall prevalence of malaria and by 2010 *P. ovale* infections were rarely reported [1]. Recently, a molecular survey found *P. ovale* in Kedougou only as mixed infections with *P. falciparum* at 11.5 % of prevalence [4].

*Plasmodium ovale* consists of two distinct species: *P. ovale curtisi* (classic type) and *P. ovale wallikeri* (variant type) [5]. The two species are indistinguishable by microscopy but seem to differ in their duration of latency [6]. The recent description of dimorphism in the gene encoding *P. ovale* tryptophan-rich antigen (*potra*) allows for the distinction between the two *P. ovale* species. Sequence and size variations were noted between the *tryptophan*-*rich antigen* genes from *P. ovale curtisi* (*poctra*) and *P. ovale wallikeri* (*powtra)* [5]. This was exploited in a nested PCR detection assay [7], where the species are discriminated by the size of the amplified fragments (299 or 317 bp for *poctra*; 245 bp for *powtra*). In addition, Tanomsing et al. developed a semi-nested PCR protocol which efficiently discriminate *P. ovale wallikeri* and *P. ovale curtisi* and found that the 299 bp fragment was overlapping between the two subspecies [8] and the 245 bp, represented *P. ovale wallikeri*.

A case of malaria due to *P. ovale wallikeri* is described in a patient admitted to the emergency unit service of the Youssou Mbargane Diop Hospital of Rufisque, a department of the peripheral region of Dakar in Senegal.

## Case presentation

A 34-year-old male baker living in Diamniadio (Dakar region) sought a consultation at a health centre near his home for fever, headache, vomiting and diarrhoea. Fever had lasted for 4 days with peaks every 48 h. The patient was suspected of malaria and was tested using an HRP-2 based RDT (detecting only *P. falciparum*). The test was negative for *P. falciparum* malaria and the patient was treated by three intravenous (IV) injection of cefotaxim (third generation of cephalosporin) at 100 mg per kg per day to cover potential bacterial infection. Owing to persisting symptoms 4 days later, he was referred to the emergency unit of the Youssou Mbargane Diop Hospital, Dakar, Senegal.

The patient had no particular previous medical history. He was born in Thies and moved to Touba at the age 12 and finally at age 23 to Diamniadio, where he has lived since. The patient claimed that he had never been out of these three areas, which are all in West Central of Senegal. Physical examination found impaired general condition but the patient was conscious. Abdomen was tender. All other physical examinations were normal. Another HRP-2 based RDT was performed and was also negative. Other laboratory tests showed anaemia (haemoglobin 11.4 g/dl), thrombocytopaenia (platelets 30 × 10^9^/mm^3^), leukopaenia (3650/mm^3^) and lymphocytopaenia (650/mm^3^). Kidney function was normal as indicated by creatininaemia and uraemia (11 mg/l and 0.13 g/l, respectively). Transaminases were slightly elevated.

**Fig. 1:**
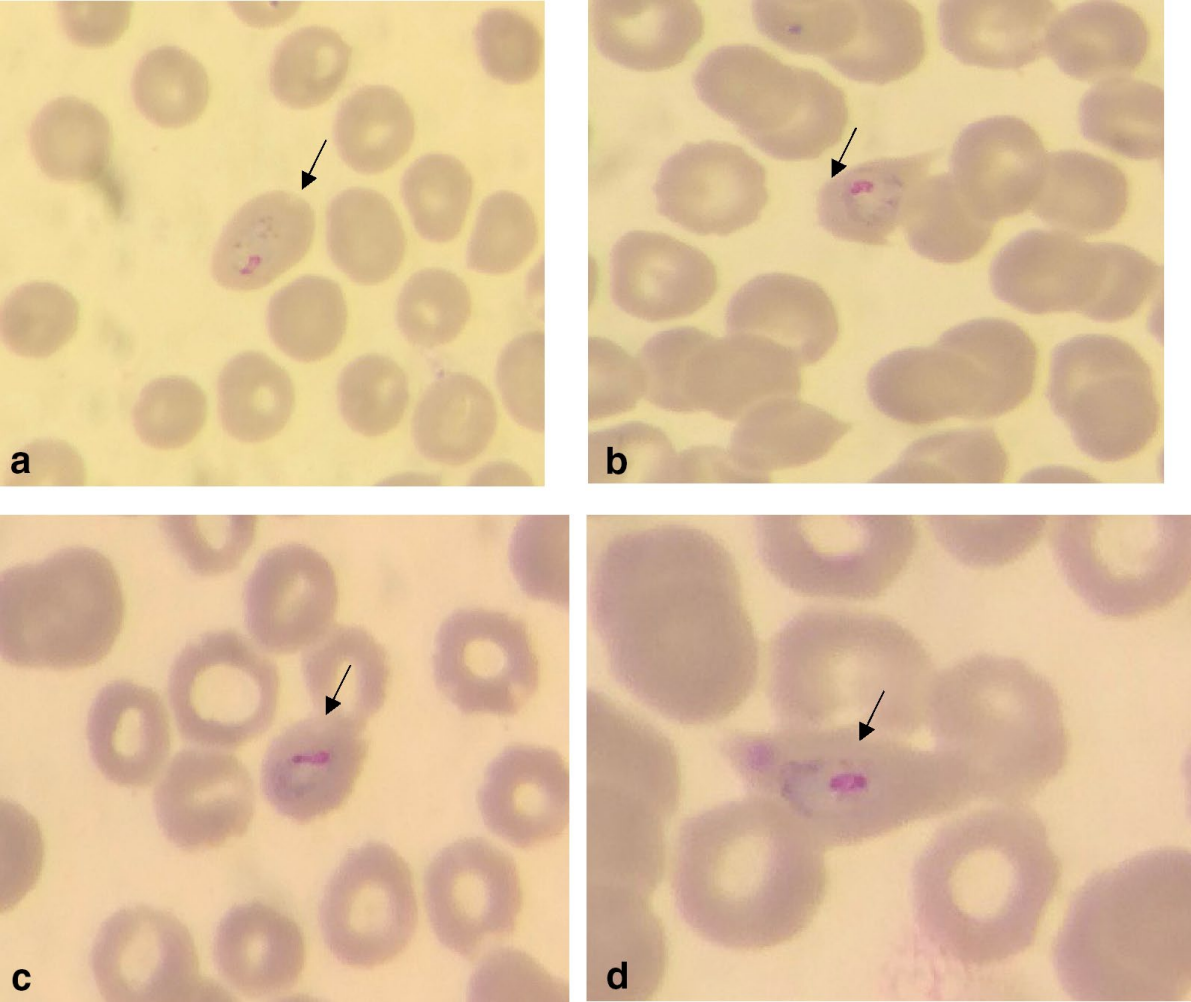
Giemsa-stained thin blood smears *Plasmodium ovale* trophozoites in enlarged and ovale shaped red blood cell (**a**) and with a fimbriated red blood cell (**b**–**d**)

Blood smear evaluations in the Parasitology Laboratory of Aristide Le Dantec University Hospital showed malaria parasites in normal and enlarged red blood cells (RBCs). Some infected RBCs were oval shaped and some were fimbriated. However, Schüffner’s dots were not observed likely due to staining defect (Fig. 1). All malaria parasite stages (rings, trophozoites, schizonts and gametocytes) were observed. These malaria parasites were identified as *P. ovale* and a 0.08 % parasitaemia was calculated. Parasite density was measured by determining the ratio of parasites/leucocytes on the basis of 8000 leucocytes/μl after counting 500 leucocytes. That ratio was found to be 1.1 giving a parasite density of 8800 parasites/μl of blood.

**Fig. 2:**
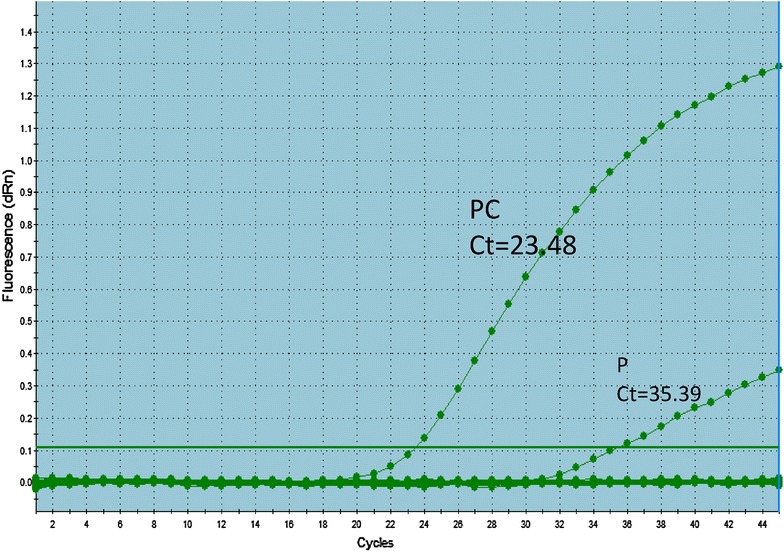
Amplification plots for the real-time PCR targeting *Plasmodium* species 18S rRNA. *P* patient sample, *PC* positive control (know *P. ovale* sample)

**Fig. 3:**
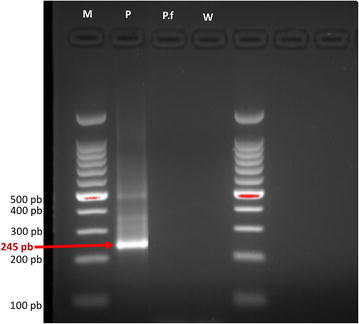
*Plasmodium ovale* subtyping nested PCR targeting *potra* gene. In nest 1, primer pair PoTRAfwd3 and PoTRArev3 bind a 787-bp fragment. Using the internal primers PoTRAfwd5 and PoTRArev5, *P. ovale wallikeri* and *P. ovale curtisi* (245 to 355 bp) can be differentiated. The 245 fragments correspond to *P. ovale wallikeri*. *P* patient sample, *W* negative control (distilled water), *Pf* negative control (*P*. *falciparum*), *M* DNA size marker

An 18S rRNA gene real-time PCR molecular assay was undertaken as previously described [9] to confirm the *P. ovale,* infection (Fig. 2). No others species of *Plasmodium* was detected. The parasite was then subtyped using the *potra* gene as target as previously described [7]. This genotyping identified the presence of *P. ovale wallikeri* (Fig. 3).

Based on clinical signs and biological findings, the patient was treated as severe malaria accordingly to the hospital’s treatment guideline. Intravenous quinine was given at admission and 24 h later. 48 h after initial treatment, oral artemether–lumefantrine was given every 6 h at six doses. On day 3 after treatment, microscopy was negative and the patient reported no further symptoms. Primaquine was not given (see “Discussion” section).

## Discussion

*Plasmodium ovale* is a malaria parasite that is endemic in sub-Saharan Africa, although not very commonly described [2]. In Senegal, little attention has been paid to *P. ovale* malaria in the recent years. Here, a case of *P. ovale* infection that appears to have been acquired in Diamniadio, near Dakar, in West Central of Senegal is reported. This case highlights the need to consider non-falciparum malaria infections in this region for case management. *Plasmodium ovale* malaria is often mild in clinical presentation [10] and severe forms of the disease are relatively rare [11–13]. The parasite densities are often less than 50 parasites/μl and a parasitaemia higher than 5000 parasites/μl is rarely reported [14]. This is believed to be due to the fact that *P. ovale* prefers younger RBCs, which are rare in peripheral blood [2]. The patient in this report presented a relatively high density parasitaemia of 8800 parasites/μl and severe thrombocytopaenia. Equally high parasitaemia levels were found in Spanish travellers infected with *P. ovale wallikeri* imported from different countries in Africa and the same study observed that severe thrombocytopaenia was more commonly associated with *P. ovale wallikeri* infection than with *P. ovale curtisi* infections [15]. While the mechanisms that produce thrombocytopaenia in malaria is still not clear it seems likely to be linked to a greater severity of the illness [16].

In Senegal, *P. ovale* subtyping is not documented. Determining the infecting *P. ovale* species may be important because recent observations suggest that the two species might differ in their relapsing patterns [6] and secondly. *Plasmodium ovale wallikeri* seems to be slightly more pathogenic [15]. The previously described dimorphisms observed in gene encoding *P. ovale* tryptophan-rich antigen (*potra*) [5] were used to identify the infecting *P. ovale* species as *P. ovale wallikeri.* This is most likely the first time *P. ovale* species determination have been undertaken when describing *P. ovale* infections in Dakar, Senegal.

The patient in this study denied having travelled outside Diamniadio suggesting that the *P. ovale* infection was likely acquired locally. Since Diamniadio is a crossroads town located at the town exit of Dakar where many different human populations from neighbouring regions and countries intersect there are two possible likely sources of the *P. ovale* parasites: (1) from an existing *P. ovale* reservoir in or near Diamniadio, or (2) that the source of the *P. ovale* infection came from another region or neighbouring countries. In the 1990s, in a study in Dielmo, exposure to *P. ovale* was reported across all ages and perennial transmission of *P. falciparum* was also noted [17], therefore, it is possible that *P. falciparum* cross immunity could protect against non-falciparum species [2]. However, in the West Central of Senegal like Diamniadio where malaria transmission is very low, this cross immunity might be low or none existing, making it easy to acquire malaria, including non-falciparum infections, as in this case report.

This present findings highlight the need to consider non-falciparum malaria infections in clinical settings both for the appropriate treatment of the infection and in order to further understand the epidemiology of malaria in the country. *Plasmodium ovale* infections present unique challenges because of the reported long latency period and the fact that two distinct species are known to exist [8]. In fact, the commonly used diagnostic tools in many endemic malaria regions, microscopy and RDT, are less sensitive when parasitaemia is very low and need expert microscopists or molecular assays to recognize uncommon species. In Senegal, *P. falciparum* is the most prevalent parasite detected when patients are suspected of malaria. Because of this, the national malaria control programme provides all health clinics of the country with HRP-2 based RDT, which are specific to *P. falciparum*. Therefore, it is possible that many cases of non-falciparum malaria infections are just not detected. This case report highlights the urgent need to develop standardized protocols capable of detecting all *Plasmodium* parasites in Senegal in order to achieve the malaria elimination agenda. In addition, although the existence of *P. ovale* hypnozoite remain controversial [18], they pose an important challenge in that they can cause relapse malaria which has great implications for treatment and transmission. Therefore, more species specific RDTs and field-usable molecular tools, such as the loop-mediated isothermal amplification (LAMP) are needed to assess the actual prevalence of *P. ovale* and other non-falciparum malaria parasites in Senegal and in other parts of Africa as previously suggested [19].

The current World Health Organization treatment guidelines recommend intravenous (IV) artesunate for the treatment of severe malaria due to all *Plasmodium* species [20]. In Senegal, since the national malaria control policies targets especially *P. falciparum*, ACT and quinine are the recommended and registered anti-malarials. The patient discussed in this case report was treated using IV quinine followed by oral ACT, which according to the current Senegalese treatment guidelines, is still the treatment of choice for severe malaria in adult patients **[**21]. ACT has shown high efficacy in the treatment of non-falciparum malaria [22]. However, a recent longitudinal study conducted in Uganda using real-time PCR demonstrates persistence of infections of both *P. falciparum* and non-falciparum infections after ACT [19]. Importantly, primaquine, which is effective in the killing of both gametocytes and hypnozoites, is currently not registered in Senegal. Availability of primaquine in the health facilities will help to provide curative treatment for hypnozoites in *P. ovale* infected patients. Currently, the national malaria control programme plans to evaluate primaquine in the north of the country in an effort to end malaria in this region [21]. Also, chloroquine, which is the standard treatment of uncomplicated non-falciparum malaria, is no longer available in Senegal. In fact, since *P. falciparum* was considered resistant to chloroquine, this drug was withdrawn and national malaria control programme did not define treatment guideline for non-falciparum malaria explaining how neglected was the non-falciparum species in Senegal [21].

## Conclusion

In summary, this case report demonstrates that *P. ovale* exists in West Central of Senegal and the prevalence is likely to be underestimated due to the diagnostic tools in place.

This case also highlights the fact that *P. ovale* malaria is not as benign as previously thought and that it is important to consider other non-falciparum malaria infections for appropriate patient care and for the success of the malaria control programme.

## Abbreviations

ACT: artemisinin-based combination therapy
HRP: histidine rich protein
LAMP: loop mediated isothermal amplification
PCR: polymerase chain reaction
*potra*: *P. ovale* tryptophan-rich antigen
RDT: rapid diagnostic test
rRNA: ribosomal ribonucleic acid
